# Associations of clock genes polymorphisms with soft tissue sarcoma susceptibility and prognosis

**DOI:** 10.1186/s12967-018-1715-0

**Published:** 2018-12-05

**Authors:** Clara Benna, Senthilkumar Rajendran, Giovanna Spiro, Saveria Tropea, Paolo Del Fiore, Carlo Riccardo Rossi, Simone Mocellin

**Affiliations:** 1 Department of Surgery Oncology and Gastroenterology, University of Padova, Padua, Italy; 2 Clinica Chirurgica I, Azienda Ospedaliera Padova, Padua, Italy; 3 Surgical Oncology Unit, Istituto Oncologico Veneto (IOV-IRCCS), Padua, Italy

**Keywords:** Soft tissue sarcoma, Circadian clock gene, Single nucleotide polymorphism (SNP), Genetic variation, Circadian pathway, Susceptibility, Risk, Prognosis, Leiomyosarcoma, Liposarcoma

## Abstract

**Background:**

Dysfunction of the circadian clock and polymorphisms of some circadian genes have been linked to cancer development and progression. We investigated the relationship between circadian genes germline variation and susceptibility or prognosis of patients with soft tissue sarcoma.

**Patients and methods:**

We considered the 14 single nucleotide polymorphisms (SNPs) of 6 core circadian genes that have a minor allele frequency > 5% and that are known to be associated with cancer risk or prognosis. Genotyping was performed by q-PCR. Peripheral blood and clinic-pathological data were available for 162 patients with liposarcoma or leiomyosarcoma and 610 healthy donors. Associations between the selected clock genes polymorphisms and sarcoma susceptibility or prognosis were tested assuming 3 models of inheritance: additive, recessive and dominant. Subgroup analysis based on sarcoma histotype was performed under the additive genetic model. Multivariate logistic regression and multivariate Cox proportional hazard regression analyses were utilized to assess the association between SNPs with patient susceptibility and survival, respectively. Pathway variation analysis was conducted employing the Adaptive Rank Truncated Product method.

**Results:**

Six out of the 14 analyzed SNPs were statistically significantly associated with susceptibility or prognosis of soft tissue sarcoma (P < 0.05). The present analysis suggested that carriers of the minor allele of the *CLOCK* polymorphism rs1801260 (C) or of *PER2* rs934945 (T) had a reduced predisposition to sarcoma (26% and 35% respectively with the additive model) and liposarcoma (33% and 41% respectively). The minor allele (A) of *NPAS2* rs895520 was associated with an increased predisposition to sarcoma of 33% and leiomyosarcoma of 44%. *RORA *rs339972 C allele was associated with a decreased predisposition to develop sarcoma assuming an additive model (29%) and leiomyosarcoma (36%). *PER1* rs3027178 was associated with a reduced predisposition only in liposarcoma subgroup (32%). rs7602358 located upstream *PER2* was significantly associated with liposarcoma survival (HR: 1.98; 95% CI 1.02–3.85; P = 0.04). Germline genetic variation in the circadian pathway was associated with the risk of developing soft tissue sarcoma (P = 0.035).

**Conclusions:**

Genetic variation of circadian genes appears to play a role in the determinism of patient susceptibility and prognosis. These findings prompt further studies to fully dissect the molecular mechanisms.

**Electronic supplementary material:**

The online version of this article (10.1186/s12967-018-1715-0) contains supplementary material, which is available to authorized users.

## Background

Circadian rhythms are approximately 24 h oscillations of biochemical, physiologic and behavioral processes, present in almost all living organisms, which arise from an ancient adaptation to the rotation of the earth. Disruption of circadian rhythms has been related to different diseases as diabetes, depression, sleep disorders, obesity, heart attack and cancer [1]. Since the eighties it has been hypothesized that the high risk of human breast and prostate cancer in industrialized societies was caused by circadian rhythms deregulation [2]. In 2007 an expert panel assembled by the International Agency for Research on Cancer (IARC) concluded that “shift-work that involves circadian disruption is probably carcinogenic to humans” [3]. The biological clock is a self-sustained mechanism able to maintain and synchronize circadian rhythms via transcription-translation feedback loops, constituted by core circadian clock genes. They can be divided in: positive activators as *CLOCK* (*clock circadian regulator*), *NPAS2* (neuronal PAS domain protein 2), *ARNTL* (aryl hydrocarbon receptor nuclear translocator like, also referred to as brain and muscle Arnt-like protein-1, *BMAL1*), *RORA* (RAR related orphan receptor A), and *NR1D1* (nuclear receptor subfamily 1 group D member 1 also known as *Rev*-*Erb alpha*); negative effectors as *CRY1* (cryptochrome circadian clock 1), *CRY2* (cryptochrome circadian clock 2), *PER1* (period circadian clock 1), *PER2* (period circadian clock 2), *PER3* (period circadian clock 3) and *NR1D2* (nuclear receptor subfamily 1 group D member 2 also known as *Rev*-*Erb beta*) and modulators as *CSNK1E* (casein kinase I epsilon). An additional clock-related gene which probably functions as modulator is *TIMELESS* (timeless circadian clock).

The circadian system is composed by the central clock, located in the suprachiasmatic nucleus of the brain and by peripheral clocks, located in virtually all body tissues. The two components communicate and synchronize with each other and, in particular, the central clock controls the peripheral clocks [4]. Moreover, the central clock modulates the expression of the so-called clock-controlled genes, which are estimated to be approximately 20% of the genes in mammals [5, 6], many of them regulating cancer-related biological pathways such as cell proliferation, apoptosis, DNA damage and repair, carcinogen metabolism and/or detoxification [7, 8].

A different approach was undertaken for the first time in 2005. Zhu and Colleagues studied a structural variant in the circadian gene PER3, which was detected to be significantly associated with increased risk of breast cancer in young women [9]. Subsequently, in a growing number of molecular epidemiological studies, germline variations in clock genes have been associated by several Authors with different type of cancer susceptibility [10] and in some cases with the prognosis of cancer patients [11–17]. Recently the discovery rate of susceptibility loci is being greatly accelerated by genome-wide association studies (GWASs) which can test up to one million single nucleotide polymorphisms (SNPs) in thousands of subjects at a time [18], nevertheless associations between clock genes variations and sarcoma susceptibility has not been explored yet.

Sarcomas are a family of rare malignant tumors arising from bone and soft tissues with more than 50 different histologies accounting for about 1–2% of cancers in adults and 15–20% in children (worldwide incidence: approximately 200,000 cases per year) [19].

Given the potential role of circadian genes in tumorigenesis, it has been hypothesized that genetic variations in these genes could be associated with an individual’s susceptibility also with sarcoma. In this exploratory analysis, we examined whether 14 common genetic variants in 6 circadian pathway genes are related to soft tissue sarcoma susceptibility or patient outcome in a series of 162 diagnosed soft tissue sarcoma cases and 610 healthy controls.

## Materials and methods

### Study design

We conducted a retrospective study to test the hypothesis that genetic variation (in terms of candidate single nucleotide polymorphisms, SNPs) of the circadian pathway might be associated to the susceptibility and the prognosis of patients affected with sarcoma. To this aim we extracted the clinico-pathological data of patients treated at our institution (University Hospital of Padova, North East of Italy) between 1992 and 2016, using a prospectively maintained database linked to our institutional biobank (Clinica Chirurgica I–Istituto Oncologico Veneto). To be included in the study, each case had to meet the following requirements: (1) histologically confirmed diagnosis of liposarcoma or leiomyosarcoma; (2) pathology-based information on TNM stage; (3) follow-up data (minimum follow up: 6 months); (4) availability of peripheral blood for genotyping purposes.

#### Patients and healthy donors

We selected retrospectively 162 consecutive sarcoma patients, 93 patients bearing liposarcoma and 69 patients bearing leiomyosarcoma, and 610 healthy controls. Healthy controls selection was both population-based (n = 270 blood donors) and hospital-based (n = 340, healthy subjects who visited the Clinica Chirurgica I ambulatories for routine check-ups). All patients signed an informed consent form explaining the research purposes of the blood withdrawal.

#### SNPs selection

We focused on 5 of the 12 core clock genes, which are CLOCK, NPAS2, PER1, PER2, RORA. Moreover, we added a clock-related gene TIMELESS, associated with cancer risk in several studies [20–23]. The CLOCK locus contains 28734 SNPs annotated by the National Center for Biotechnology Information (NCBI) (https://www.ncbi.nlm.nih.gov/) SNP data repository. The Genome Variation Server of the University of Washington (http://gvs.gs.washington.edu/GVS/) and the TagSNP tool of the US National Institute of Environmental Health Sciences (https://snpinfo.niehs.nih.gov/snpinfo/snptag.html) were interrogated to identify the SNPs that tag CLOCK SNPs with a minimum correlation coefficient (r^2^) of at least 0.80, minimal genotype data coverage of 50%, and minimal allele frequency of 5%, in the Caucasian population (CEU) genotyped by the HapMap Project. This process yielded an initial group of 134 Tag SNPs for genotyping, divided in 11 bins (groups of SNPs in high linkage disequilibrium, D’ greater than 0.8). Excluding those SNPs tagging less than 10 SNPs, we selected from each bin the Tag SNPs with more functional/cancer related information available. They are the following: rs1801260, rs3736544, rs3749474. Moreover, we included rs34897046 which has a missense functional effect.

The TIMELESS locus contains 9200 SNPs annotated by the NCBI dbSNP. With the same procedure described, rs774027 was chosen as TagSNP. In order to enrich our analysis, rs3809125 (3′-UTR) and rs7302060 were added based on literature (see Table 2) [20].

For NPAS2, PER1, PER2, RORA we decided to follow a different approach. Despite the elevated number of Tag SNPs for each gene (more than 200 in NPAS2 locus) none of them tags more than 9 SNPs and most of them tags 1 SNP. We relied on our previous meta-analysis [10] or on literature (see Table 2).

NPAS2 rs895520, PER2 rs7602358, RORA rs339972, and rs10519097 had a statistically significant association with cancer risk. Moreover, regarding the NPAS2 locus, we selected the most studied variant, based on number of datasets, with missense functional effect, rs2305160. PER1 rs3027178 and PER2 rs934945 were selected based on literature [24–28].

### DNA extraction and genotyping

#### DNA extraction

Genomic DNA was isolated from peripheral whole blood employing the QIAamp DNA Mini Kit (Qiagen, Hilden, Germany) following the manufacturer’s instructions and quantified by Nanodrop 1000 spectrophotometer (Thermofisher Scientific, Waltham, MAUSA).

#### Subjects’ genotyping

10 to 20 ng of DNA of each patient were used for TaqMan SNP genotyping assays (comprising primers and fluorescent probes) according to the manufacturer’s instructions (Thermofisher Scientific, Waltham, MA USA). Genotyping was performed by real–time PCR using either allelic discrimination in the 7300 RT–PCR System (Thermofisher Scientific, Waltham, MA USA), either using endpoint genotyping in an LightCycler 480II (Roche Molecular Diagnostics Pleasanton, CA, USA). For the 7300 RT–PCR System PCR parameters involved an initial denaturation at 95 °C for 10 min followed by 40 cycles at 95 °C for 15 s and 60 °C for 1 min. Post run data were analysed by 7300 SDS software (Thermofisher Scientific, Waltham, MA USA) and Automatic calls were assigned with approximately 99.8% quality. A call rate > 95% was considered the cutoff to consider genotyping. For LightCycler 480II PCR parameters involved an initial denaturation at 95 °C for 10 min followed by 40 cycles at 95 °C for 10 s, 60 °C for 1 min, and 72 °C for 10 s. Post run data were analysed by LightCycler 480 Endpoint Genotyping software (Roche Molecular Diagnostics Pleasanton, CA, USA). Two blank (water) controls in each 96-well plate were used for the assay quality control.

### Statistical analysis

We considered three genetic models:

1. Additive (or allelic; presence of 0 vs 1 vs 2 copies of the variant allele): the genotype for each SNP is considered as a continuous variable;
2. Recessive (presence of 2 copies of the variant allele vs 0 or 1 copies of the variant allele): the genotype for each SNP is considered as a categorical variable with 2 categories (homozygous for the common allele + heterozygous, homozygous for the variant allele);
3. Dominant (presence of at least one copy of the variant allele vs no copies of the variant allele): the genotype for each SNP is considered as a categorical variable with 2 categories (homozygous for the variant allele + heterozygous, homozygous for the common allele).

For susceptibility of sarcoma assessment multivariate logistic regression was employed for each genetic model. Odds ratios (OR) and 95% confidence intervals were used as a measure of association. In those multivariate models the evaluated outcome was the presence or absence of sarcoma, while the explanatory variables were the single SNPs adjusted for age and gender.

For prognosis of sarcoma assessment multivariate Cox proportional hazard regression was employed. Overall survival was defined as the time from the date of tumor diagnosis to the date of death by any cause or last follow-up visit. Hazard ratios (HR) and 95% confidence intervals were used as a measure of association. In those multivariate models the evaluated event was the patient’s survival, the time to event were the months of survival, and the explanatory variables were the single SNP adjusted for age, gender and sarcoma stage.

For discriminating whether sarcoma subtype interacts with SNP associations with susceptibility or prognosis, the above mentioned statistical analyses were repeated, for the liposarcoma subgroup and for the leiomyosarcoma subgroup, employing the additive model because the additive model can be considered a conservative choice between recessive and dominant models.

Hardy–Weinberg equilibrium (HWE) was tested for both samples (patients and healthy controls) for each SNP employing OEGE—Online Encyclopedia for Genetic Epidemiology studies [29], http://www.oege.org/software/hwe-mr-calc.shtml. This tool is a HWE calculator for biallelic SNPs based on Chi-square statistic.

Statistical power was calculated for each SNP employing the on-line tool “Power and Sample Size” of the University of Vanderbilt (http://biostat.mc.vanderbilt.edu/wiki/Main/PowerSampleSize) [30]. Power was defined as the probability of correctly rejecting the null hypothesis that the relative risk (OR) was equal to 1, given 162 case patients and 610 controls. The type I error probability α was set to 0.05 and ψ (OR considered clinically relevant in our study) was set to 0.80.

For all the analysis conducted in this study, a P value < 0.05 was used as the cut-off for significance, not adjusted for multiple comparisons.

Rcmdr: R Commander. R package version 2.4-2 was employed for the analyses.

#### Pathway variation analysis

We also investigated the associations of soft tissue sarcoma with genes, observed as combinations of SNPs, and with the circadian rhythm pathway as a whole, observed as combinations of 6 clock genes. This analysis was conducted employing the Adaptive Rank Truncated Product (ARTP) method [31] as we already described in detail in [32].

## Results

The analysis was based on a total of 162 cases of sarcoma and 610 healthy controls, all of European ancestry. Subjects’ characteristics and the main features of the SNPs we investigated are summarized in Tables 1 and 2, respectively.

**Table 1:** Subjects’ characteristics of sarcoma cases and healthy controls retrospectively enrolled in this study

Characteristic	Controls (n = 610)	Cases (n = 162)	Liposarcoma (n = 93, 57.4%)	Leiomyosarcoma (n = 69, 42.6%)
Mean age, years (st.dev.)	48.6 (14.8)	60.0 (14.0)	59.8 (13.3)	60.2 (14.9)
Gender, n (%)				
Male	336 (55.2)	95 (58.6)	65 (69.9)	30 (43.5)
Female	273 (44.8)	67 (41.4)	28 (30.1)	39 (56.5)
Source of controls, n (%)				
Hospital	340 (55.7)			
Population	270 (44.3)			
Patient status, n (%)				
Alive		89 (54.9)	66 (71.0)	23 (33.3)
Deceased		73 (45.1)	27 (29.0)	46 (66.7)
Median survival, months (min, max)		79.0 (1.0, 366.0)	91.5 (2.0, 366.0)	42.0 (1.0, 196.0)
Tumoral stage, n (%)				
I		37 (23.0)	23 (25.0)	14 (20.3)
II		54 (33.5)	36 (39.1)	18 (26.1)
III		55 (34.2)	29 (31.5)	26 (37.7)
IV		15 (9.3)	4 (4.3)	11 (15.9)

**Table 2:** SNPs main features of circadian genes tested in this study

Gene	SNP ID	Genotype	Ctrls	Cases	P-val.	Chr	Region	Residue	Ref in cancer
*CLOCK*	rs1801260	TT	323	92	0.21	4	3′-UTR		[10, 12, 13, 15, 16, 25, 27, 33, 34]
		TC	228	62					
		CC	56	8					
	rs3736544	GG	241	60	0.72	4	Exon	Asn>Asn	
		GA	269	78					
		AA	95	24					
	rs3749474	CC	259	62	0.45	4	3′-UTR		[10, 15, 16, 33, 35]
		CT	266	80					
		TT	83	20					
	rs34897046	GG	566	149	0.60	4	Exon	Ser>Cys	
		GC	40	13					
		CC	1	0					
*NPAS2*	rs895520	GG	211	49	0.20	2	Intron		[10, 26, 27, 36–38]
		GA	294	76					
		AA	103	37					
	rs2305160	GG	283	75	1.00	2	Exon	Thr>Ala	[10, 14, 15, 21, 23, 26, 27, 34–45]
		GA	264	71					
		AA	59	16					
*PER1*	rs3027178	TT	281	84	0.29	17	Exon	Thr>Thr	[10, 17, 28, 46]
		TG	253	64					
		GG	76	14					
*PER2*	rs934945	CC	386	118	0.07	2	Exon	Gly>Glu	[10, 17, 24–28, 36, 37, 46]
		CT	206	42					
		TT	17	2					
	rs7602358	TT	358	87	0.49	2	Intron		[10, 23, 27, 36–38, 40]
		TG	213	64					
		GG	38	11					
*RORA*	rs339972	TT	312	97	0.15	15	Intron		[10, 27, 36, 37]
		TC	233	53					
		CC	62	12					
	rs10519097	CC	422	110	0.67	15	Intron		[10, 27, 36, 37]
		CT	173	50					
		TT	14	2					
*TIMELESS*	rs774027	AA	157	44	0.63	12	Exon	Ile > Val	[10, 23, 27, 38]
		AT	301	74					
		TT	148	44					
	rs3809125	CC	250	70	0.87	12	Upstream		
		CT	280	71					
		TT	74	20					
	rs7302060	TT	181	55	0.53	12	Intron		[10, 20–22]
		TC	304	74					
		CC	121	33					

*Ctrl* controls, *P-val* P value, *Chr* chromosome, *Region* Gene region, *Residue* residue change, *Ref in cancer* reference in the literature regarding cancer associations

A total of 14 preselected SNPs in 6 circadian clock genes were successfully genotyped, and no departures from Hardy–Weinberg equilibrium were observed neither among the controls nor among the patients, as reported in Additional file 1: Table S1 (P > 0.05). The *CLOCK* rs34897046 SNP showed minor allele frequencies < 5% across the whole study population (3% in controls and 4% in cases, see Additional file 1: Table S1) and was not evaluated further statistically.

Statistical power for each SNP for primary analysis (sarcoma) and subgroup analysis (liposarcoma and leiomyosarcoma) is reported in Additional file 2: Table S2.

### Susceptibility assessment

Associations between the selected clock genes polymorphisms and sarcoma predisposition were tested assuming 3 models of inheritance: additive, recessive and dominant. We used odds ratios (ORs) and their corresponding 95% confidence intervals (95% CI) to measure the strength of association between each polymorphism and sarcoma risk. The results are reported in Table 3. We examined further subgroup SNP associations according to sarcoma histology: liposarcoma (n = 93) and leiomyosarcoma (n = 69). The results are reported in Table 4.

**Table 3:** Associations of circadian pathway genes with predisposition to sarcoma under 3 models of inheritance: additive, recessive, dominant

Gene	SNP ID	Minor allele	Additive		Recessive		Dominant	
			OR [95% CI]	P-val.	OR [95% CI]	P-val.	OR [95% CI]	P-val.
*CLOCK*	*rs1801260*	C	*0.74 [0.55–1.00]*	*0.05*	*0.40 [0.18–0.88]*	*0.02*	0.79 [0.54*–*1.14]	0.20
	rs3736544	A	1.15 [0.89*–*1.50]	0.29	1.10 [0.66*–*1.83]	0.72	1.27 [0.87*–*1.86]	0.22
	rs3749474	T	1.06 [0.81*–*1.38]	0.68	0.94 [0.54*–*1.62]	0.82	1.15 [0.79*–*1.67]	0.47
*NPAS2*	*rs895520*	A	*1.33 [1.02–1.73]*	*0.03*	*1.70 [1.08–2.68]*	*0.02*	1.30 [0.87*–*1.93]	0.20
	rs2305160	A	0.95 [0.72*–*1.26]	0.71	0.97 [0.52*–*1.79]	0.92	0.92 [0.64*–*1.34]	0.68
*PER1*	rs3027178	G	0.80 [0.61*–*1.06]	0.12	0.72 [0.39*–*1.35]	0.31	0.76 [0.53*–*1.10]	0.15
*PER2*	*rs934945*	*T*	*0.65 [0.45–0.94]*	*0.02*	0.48 [0.10*–*2.25]	0.35	*0.63 [0.42–0.95]*	*0.03*
	rs7602358	G	1.21 [0.90*–*1.62]	0.21	1.18 [0.56*–*2.48]	0.66	1.29 [0.89*–*1.86]	0.18
*RORA*	*rs339972*	*C*	*0.71 [0.53–0.96]*	*0.02*	0.69 [0.35*–*1.36]	0.28	*0.64 [0.44–0.93]*	*0.02*
	rs10519097	T	0.98 [0.68*–*1.40]	0.89	0.55 [0.12*–*2.53]	0.44	1.02 [0.69*–*1.51]	0.93
*TIMELESS*	rs774027	T	0.98 [0.76*–*1.26]	0.85	1.02 [0.67*–*1.55]	0.92	0.92 [0.61*–*1.39]	0.69
	rs3809125	T	0.92 [0.70*–*1.20]	0.52	0.89 [0.51*–*1.55]	0.67	0.89 [0.62*–*1.30]	0.55
	rs7302060	C	0.98 [0.76*–*1.27]	0.89	1.03 [0.65*–*1.62]	0.91	0.94 [0.63*–*1.39]	0.75

Italic values indicate statistically significant associations (P < 0.05)

*OR [95% CI]* Odds Ratio [95% Confidence Interval], *P-val*. P-value

**Table 4:** Association of circadian pathway genes with predisposition to liposarcoma and leiomyosarcoma in subgroups analysis under the additive genetic model

Gene	SNP ID	*Liposarcoma*		*Leiomyosarcoma*	
		OR [95% CI]	P-val.	OR [95% CI]	P-val.
*CLOCK*	*rs1801260*	*0.67 [0.46–0.98]*	*0.04*	0.84 [0.56*–*1.25]	0.39
	rs3736544	1.23 [0.88*–*1.71]	0.22	1.09 [0.76*–*1.57]	0.64
	rs3749474	1.09 [0.78*–*1.53]	0.60	1.00 [0.69*–*1.46]	0.98
*NPAS2*	rs895520	1.26 [0.90*–*1.75]	0.18	*1.44 [0.99–2.09]*	*0.05*
	rs2305160	1.01 [0.72*–*1.43]	0.95	0.88 [0.59*–*1.32]	0.54
*PER1*	*rs3027178*	*0.68 [0.47–0.98]*	*0.04*	0.96 [0.66*–*1.41]	0.85
*PER2*	*rs934945*	*0.59 [0.37–0.96]*	*0.03*	0.71 [0.42*–*1.20]	0.20
	rs7602358	1.23 [0.85*–*1.77]	0.28	1.16 [0.77*–*1.75]	0.49
*RORA*	rs339972	0.75 [0.52*–*1.07]	0.12	*0.64 [0.42–0.98]*	*0.04*
	rs10519097	0.82 [0.51*–*1.31]	0.40	1.28 [0.80*–*2.06]	0.31
*TIMELESS*	rs774027	0.98 [0.71*–*1.35]	0.90	0.95 [0.66*–*1.35]	0.76
	rs3809125	0.99 [0.71*–*1.38]	0.95	0.79 [0.53*–*1.16]	0.23
	rs7302060	1.00 [0.72*–*1.37]	0.97	1.01 [0.70*–*1.45]	0.97

Italic values indicate statistically significant associations (P < 0.05)

*OR [95% CI]* Odds Ratio [95% Confidence Interval], *P-val*. P-value

*CLOCK* maps on chromosome 4 at 4q12. CLOCK putative protein belongs to the basic helix-loop-helix PAS family of transcription factors and forms heterodimers with ARNTL (BMAL1) to enhance target gene expression. CLOCK is also involved in growth arrest, DNA repair and apoptosis upon genotoxic stress caused by UV radiation, suggesting that this molecule may represent an important “caretaker” promoting cell cycle arrest upon DNA damage [47]. CLOCK has the properties of a histone acetyl transferase and is involved in chromatin remodeling [48].

rs1801260 is located on 3′-UTR region of *CLOCK*. Carriers of the minor allele (C) rs1801260 had a reduced predisposition to sarcoma under an additive (per allele OR 0.74; 95% CI 0.55–1.00; P = 0.05) and a recessive (OR 0.40; 95% CI 0.18–0.88; P = 0.02) genetic model. Subgroup analysis suggested that the protective effect of the C allele was significantly associated with liposarcoma under an additive model (per allele OR 0.67; 95% CI 0.46–0.98; P = 0.04). No significant association was found with leiomyosarcoma.

*NPAS2* is the largest human clock gene. It maps on chromosome 2 at 2q11.2 and, like its paralogue *CLOCK*, encodes for a member of the basic helix-loop-helix PAS class of transcription factors [49]. When dimerized with ARNTL, NPAS2 binds to E-box regulatory elements in target promoter regions and enhances target gene expression. Previous studies reported NPAS2 as a putative tumor suppressor [50]. rs895520 is located on an intron of *NPAS2* locus. The present analysis suggested that the minor allele (A) was associated with an increased predisposition to sarcoma of 33% under an additive (per allele OR 1.33; 95% CI 1.02–1.73; P = 0.03) and a recessive (OR 1.70; 95% CI 1.08–2.68; P = 0.02) model. In subgroup analysis this association was statistically significant in leiomyosarcoma (per allele OR 1.44; 95% CI 0.99–2.09; P = 0.05).

*PERs* code for PAS domain-containing key regulators of the circadian clock. *PER* genes control their own transcription by directly repressing ARNTL heterodimers, their activators [49]. Moreover, it has been suggested that PER1 and PER2 function as tumor suppressors [51–56]. *PER2* gene can activate c-Myc signaling pathways leading to genomic instability and cell proliferation. PER2 dysfunction can also impair p53-mediated apoptosis and consequently result in genomic instability and the accumulation of damaged cells [55, 57]. *PER1* maps on chromosome 17 at 17p13.1 and *PER2* on chromosome 2 at 2q37.3.

rs934945 (C>T) is located on the last exon of PER2 locus and has a missense functional effect, leading to the substitution Glycine–Glutamic Acid. Carriers of the minor allele had a decreased predisposition to develop sarcoma (35%) employing an additive genetic model (per allele OR 0.65; 95% CI 0.45–0.94; P = 0.02) or a dominant model and (OR 0.63; 95% CI 0.42–0.95; P = 0.03). This association is statistically significant also in liposarcoma subgroup (per allele OR 0.59; 95% CI 0.37–0.96; P = 0.03). Moreover, in liposarcoma subgroup *PER1* rs3027178, a genetic variant with a synonymous functional effect, was associated with a reduced predisposition (per allele OR 0.68; 95% CI 0.47–0.98; P = 0.04).

*RORA* maps on chromosome 15 at 15q21-q22, spans a 730 kb large genomic region comprised of 15 exons and encodes for one member of the retinoid orphan nuclear receptor subfamily of orphan receptors. RORA has been reported as potential tumor suppressor [58, 59]. ARNTL-CLOCK or ARNTL-NPAS2 heterodimers promote the transcription of *RORA*, which in turn activates the transcription of *ARNTL.*

rs339972 C allele was associated with a decreased predisposition to develop sarcoma assuming an additive (per allele OR 0.71;95% CI 0.53–0.96; P = 0.02) or a dominant (OR 0.64; 95% CI 0.44–0.93; P = 0.02) genetic model. This association is statistically significant in leiomyosarcoma subgroup (per allele OR 0.64; 95% CI 0.42–0.98; P = 0.04).

No significant associations were found for the selected *TIMELESS* SNPs and the predisposition to develop sarcoma.

### Prognosis assessment

rs7602358 located upstream PER2 was significantly associated with liposarcoma survival (HR 1.98; 95% CI 1.02–3.85; P = 0.04) employing an additive genetic model. Carriers of the minor allele (G) had an increased risk of mortality of 98%. No further statistically significant associations with prognosis were found, neither in primary analysis nor in subgroup analysis. Results are reported in Additional file 3: Table S3.

### Pathway variation analysis

The results of the pathway variation analysis are reported in Table 5. We found a significant association between circadian pathway variation and risk of developing this tumour (circadian pathway *P* value 0.035). This result was based on the data regarding 12 SNPs located in six genes. The top genes were *PER2* (2 SNPs, circadian gene *P* value 0.036) and RORA (2 SNPs, circadian gene *P* value 0.050).

**Table 5:** Pathway variation analysis

Gene	Chr N	SNP	P-value
*PER2*	2	2	0.036
*RORA*	15	2	0.050
*NPAS2*	2	2	0.068
*PER1*	17	1	0.120
*CLOCK*	4	2	0.504
*TIMELESS*	12	3	0.814
PATHWAY		12	0.035

## Discussion

### Summary

In the present article we described the findings of the first study, to our knowledge, investigating the relations between circadian clock genes DNA genetic variations and the susceptibility to soft tissue sarcoma or the outcome of sarcoma bearing patients. The results were based on genotyping data from 772 people enrolled retrospectively with a control-to-case ratio equal to 3.

We hypothesized that circadian clock genes SNPs may influence the susceptibility to soft tissue sarcoma as suggested for many different types of cancer as breast, prostate, colorectal, ovarian, pancreatic, lung, glioma, and non-Hodgkin lymphoma [10]. Our results argue in favor of this hypothesis, in fact 4 of the 13 analyzed genetic variant were statistically significantly associated with the predisposition to sarcoma employing an additive model of inheritance. They are: *CLOCK *rs1801260, *NPAS2 *rs895520, *PER2 *rs934945, and *RORA* rs339972. In our previous meta-analysis on sarcoma genetic variations [60] we highlighted the interesting fact that the susceptibility, defined as odds ratio, statistically significantly associated with single variants ranged between higher values (mean approximately 1.70) than those usually observed for other malignancies such as breast [61], colorectal [62], and gastric carcinomas [63], which generally include odds ratios between 1.10 and 1.30. The present study is in line with this observation (mean approximately 1.40) fueling the speculation that germline DNA variation is especially important in the determinism of the predisposition to this family of tumors.

### Susceptibility to soft tissue sarcoma

*CLOCK* rs1801260, located on 3′-UTR region, was studied by many Authors in relation to breast cancer [27, 33, 34], colorectal cancer [15, 25], esophageal carcinoma [12, 13], and gastric cancer [16]. Our previous meta-analysis on genetic variation of clock genes and cancer risk [10] failed to reveal an association with cancer risk. Primary meta-analysis relied upon 4 datasets, including 2578 cases and 3349 controls, 3 out of 4 were based on breast cancer patients. Stratified analysis by cancer type showed lack of association with breast cancer. The fourth dataset was retrieved by the publication of Karantanos and Colleagues [25] on colorectal cancer. Karantanos found that the minor allele (C) and the CC genotype of rs1801260 polymorphism conferred an increased risk (78%) for the CRC development. Our findings in the present study showed that carriers of the C allele of rs1801260 had a reduced predisposition to sarcoma (26%).

*CLOCK* rs1801260 was considered by Authors also in relation to outcome. According to a study by Zhou et al. [15] who evaluated the association between clock genes polymorphisms and prognosis in patients with colorectal cancer, the presence of T allele and the TT genotype of rs1801260 SNP were related to decreased overall survival and unfavorable prognosis. In gastric cancer our group [16] suggested that rs1801260 C allele affect patient survival only if combined with the major allele of *CLOCK* rs3749474. In the present study we found no association with patients’ prognosis.

Considering all these observations one may speculate that the C allele effect is strongly dependent on the tumor site.

*NPAS2 *rs895520 is located on an intron of *NPAS2* locus. Our previous meta-analysis of four datasets (including 19,865 subjects) revealed a highly significant association with an intermediate level of evidence with cancer risk (summary OR 1.08; 95% CI 1.03–1.13; P = 0.001). This result is also congruent with findings from a NCI genome-wide association study on prostate cancer (CGEMS project), released in 2006 and not included in our meta-analysis, that showed some significant associations between variants in circadian genes and prostate cancer susceptibility. The CGEMS project genotyped 550,000 SNPs in 1182 prostate cancer cases and 1174 controls [64]. A total of 104 SNPs in circadian genes were included. Eight of these, located in four genes, NPAS2 (2 SNPs), CSNK1E (3 SNPs), CRY1 (2 SNPs), and CRY2 (1 SNP), were significantly associated with prostate cancer risk. NPAS2 rs895521 was associated with P ≤ 0.01. The present analysis suggested that the minor allele (A) was associated with an increased predisposition to sarcoma of 33%.

*PER2 *rs934945 has a missense functional effect causing glycine to glutamic acid substitution in PER2 protein and is probably related to a decreased PER2 activity. Dai and Colleagues [24] suggested that individuals carrying both the *CLOCK* rs3805151 (not considered in our study) CC and *PER2* TT genotype had an increased breast cancer risk (OR 2.28; 95% CI 1.22–4.26). In the current study we evaluated the association between this polymorphism and the predisposition to develop sarcoma and we found that carriers of the minor allele (T) had a decreased risk (35%). As for *CLOCK* the effect could be attributed to a different susceptibility depending on the cancer type.

*RORA *rs339972 is located on an intron of *RORA* locus. In our previous meta-analysis this SNP was found to be associated to the risk of cancer (summary OR 1.08; 95% CI 1.01–1.15; P = 0.02). Primary meta-analysis relied on 2 datasets of breast cancer patients and one of pancreatic cancer. In the current study rs339972 C allele was associated with a decreased predisposition to develop sarcoma (per allele OR 0.71; 95% CI 0.53–0.96; P = 0.02).

### Liposarcoma and leiomyosarcoma subgroups

Stratification by sarcoma histotype showed also potentially interesting results. Employing an additive model of inheritance *CLOCK* rs1801260 and *PER2* rs934945 were statistically significantly associated with liposarcoma, while *NPAS2* rs895520 and* RORA *rs339972 were statistically significantly associated with leiomyosarcoma. Moreover, *PER1* rs3027178 was found to be associated only with liposarcoma susceptibility. It can be argued that different circadian clock genes influence specifically a particular sarcoma subtype. NPAS2 is a paralog of CLOCK. Both proteins are major regulators of the molecular clock, and act by forming heterodimers with ARNTL and promoting transcription of target genes. However, while *CLOCK* is mainly expressed in the ‘‘central pacemaker’’ of the circadian system, the suprachiasmatic nucleus of the hypothalamus, *NPAS2* is expressed mainly in the forebrain [65]. This suggests that while these 2 genes are functionally analogous, they might be involved in separate circadian-controlled processes. The circadian clock directs nearly all aspects of diurnal physiology, including lipid metabolism and fat cell differentiation [66–68]. Recently, *CLOCK* and *PER2* polymorphisms have been linked to obesity and the metabolic syndrome [69, 70]. Since it has been proposed that liposarcoma could arise from the dedifferentiation of fat cells [71], one could hypothesize a specific role of *CLOCK* and *PER2* genes and this aspect, worth to be investigated, could open the avenue to new therapeutic targets.

### Prognosis

Genetic variation in the circadian system has been associated with tumor aggressiveness or patient survival in hepatocellular carcinoma [28, 46], melanoma [72] as well as prostate [11], colorectal [15], gastric [16, 17] and breast [14] cancer. The present results also suggest a potential impact of circadian clock on liposarcoma survival. In particular, rs7602358 located upstream *PER2*, was significantly associated with liposarcoma prognosis. rs7602358 was previously considered by four research groups [23, 27, 38, 40] evaluating the associations with prostate cancer, breast cancer, and glioma risk. Zhu and Colleagues [23] found a difference in risk association of rs7602358 with prostate cancer between less aggressive and more aggressive prostate cancer subgroup, leading to suppose a role of *PER2* in malignant cells aggressiveness.

### Pathway variation analysis

We used a gene- and pathway-based approach to investigate the overall effect of circadian clock gene germline variations on soft tissue sarcoma risk. This approach is useful to detect the combined effects of genetic polymorphisms that are weakly associated with the disease but may not be detected in single-SNP analyses and may provide additional insights into the mechanisms underlying disease susceptibility. Our results suggest that genetic variation in the circadian rhythm pathway as a whole is related to soft tissue sarcoma susceptibility. This association was mostly driven by PER2 and RORA. To the best of our knowledge, this is the first time that ARTP-based gene and pathway analysis has been applied to the relationship between circadian genes’ germline variation and soft tissue sarcoma susceptibility.

### Strength and limitations

Our study is notable for several strengths. To the best of our knowledge, this is the first study investigating the associations of germline circadian clock genes polymorphisms in relation to risk or prognosis of soft tissue sarcoma. Given the rarity of sarcoma, we leveraged the existing resources of our biobank to evaluate a relatively large cohort of patients with available germline DNA. We used several strategies to select the potentially interesting variations: by using tag SNPs, we were able to efficiently interrogate multiple regions of the *CLOCK* and *TIMELESS* genes.

Our study is also limited by a number of weaknesses. Due to sample size considerations and to our resources, we limited the number of circadian clock genes selected and SNPs evaluated. We considered three genetic models of inheritance without correcting for multiple testing. Any multiple testing correction would probably nullify statistical significance, nevertheless the strength of those association is noticeably higher than that usually observed for other tumor types. However, we considered this work a starting point and we did not know a priori the best fitted model. The power of most of our comparisons is around 30–40% and a larger sample would be needed to reach the commonly used statistical power of 80%. We pooled together different sarcoma histotypes to assess whether there are associations linked to soft tissue sarcoma in general but, given the complexity of this pathology, this could be considered a pitfall. In this regard we performed subgroup analysis and in fact we found that potentially histotype interacts with different SNPs. The source of controls is mixed, population and hospital based, nevertheless to avoid selection biases for the hospital based fraction we chose patients from different conditions.

## Conclusions

In conclusion, the present work represents the first article on association between genetic variation of clock-related genes and soft tissue sarcoma susceptibility or prognosis. Our findings support this relationship and might become a useful starting point for future investigation, which is certainly needed to shed more light in this promising field of cancer predisposition.

## Additional files

**Additional file 1: Table S1.** Hardy-Weimberg equilibrium (HWE) assumption test for all the SNPs analyzed in this study in healthy controls and sarcoma patients.

**Additional file 2: Table S2.** Statistical power for all the SNPs tested in this study in sarcoma patients.

**Additional file 3: Table S3.** Upper panel: associations of circadian pathway genes with prognosis of sarcoma under 3 models of inheritance (additive, recessive, dominant); lower panel: association of circadian pathway genes with prognosis of liposarcoma and leiomyosarcoma in subgroups analysis under the additive genetic model.
