# Supplementary material for: Associations of clock genes polymorphisms with soft tissue sarcoma susceptibility and prognosis
Source: J Transl Med. 2018 Dec 5;16:338. doi: 10.1186/s12967-018-1715-0 (PMC6280400; doi:10.1186/s12967-018-1715-0)
Supplement: Supplementary file 1 — Additional file 1: Table S1. Hardy-Weimberg equilibrium (HWE) assumption test for all the SNPs analyzed in this study in healthy controls and sarcoma patients. [file 12967_2018_1715_MOESM1_ESM.doc]

**Additional file 1: Table S1** *Hardy-Weimberg equilibrium (HWE) assumption test for all the SNPs analyzed in this study in healthy controls and sarcoma patients.*

|  |  |  | ***Controls*** | | | | ***Cases*** | | | |
| --- | --- | --- | --- | --- | --- | --- | --- | --- | --- | --- |
| ***Gene*** | **SNP ID** | **Genotype** | **Expected** | **Observed** | ***X^2^*** | **P-val.** | **Expected** | **Observed** | ***X*^2^** | **P-val.** |
| ***CLOCK*** | **rs1801260** | TT | 314.61 | 323 | 2.85 | 0.09 | 93.39 | 92 | 0.36 | 0.55 |
|  |  | TC | 244.78 | 228 |  |  | 59.22 | 62 |  |  |
|  |  | CC | 47.61 | 56 |  |  | 9.39 | 8 |  |  |
|  | **rs3736544** | GG | 233.06 | 241 | 1.88 | 0.17 | 60.5 | 60 | 0.03 | 0.86 |
|  |  | GA | 284.88 | 269 |  |  | 77 | 78 |  |  |
|  |  | AA | 87.06 | 95 |  |  | 24.5 | 24 |  |  |
|  | **rs3749474** | CC | 252.74 | 259 | 1.23 | 0.27 | 64.22 | 62 | 0.56 | 0.45 |
|  |  | CT | 278.53 | 266 |  |  | 75.56 | 80 |  |  |
|  |  | TT | 76.74 | 83 |  |  | 22.22 | 20 |  |  |
|  | **rs34897046** | GG | 565.73 | 566 | 0.11 | 0.74 | 149.26 | 149 | 0.28 | 0.60 |
|  |  | GC | 40.55 | 40 |  |  | 12.48 | 13 |  |  |
|  |  | CC | 0.73 | 1 |  |  | 0.26 | 0 |  |  |
| ***NPAS2*** | **rs895520** | GG | 210.8 | 211 | 0 | 1 | 46.72 | 49 | 0.52 | 0.47 |
|  |  | GA | 294.41 | 294 |  |  | 80.56 | 76 |  |  |
|  |  | AA | 102.8 | 103 |  |  | 34.72 | 37 |  |  |
|  | **rs2305160** | GG | 284.2 | 283 | 0.05 | 0.82 | 75.37 | 75 | 0.02 | 0.89 |
|  |  | GA | 261.6 | 264 |  |  | 70.26 | 71 |  |  |
|  |  | AA | 60.2 | 59 |  |  | 16.37 | 16 |  |  |
| ***PER1*** | **rs3027178** | TT | 272.22 | 281 | 2.57 | 0.11 | 83.06 | 84 | 0.13 | 0.72 |
|  |  | TG | 270.55 | 253 |  |  | 65.88 | 64 |  |  |
|  |  | GG | 67.22 | 76 |  |  | 13.06 | 14 |  |  |
| ***PER2*** | **rs934945** | CC | 392.65 | 386 | 2.9 | 0.09 | 119.27 | 118 | 0.67 | 0.41 |
|  |  | CT | 192.71 | 206 |  |  | 39.47 | 42 |  |  |
|  |  | TT | 23.65 | 17 |  |  | 3.27 | 2 |  |  |
|  | **rs7602358** | TT | 354.29 | 358 | 0.69 | 0.41 | 87.41 | 87 | 0.03 | 0.86 |
|  |  | TG | 220.43 | 213 |  |  | 63.17 | 64 |  |  |
|  |  | GG | 34.29 | 38 |  |  | 11.41 | 11 |  |  |
| ***RORA*** | **rs339972** | TT | 302.49 | 312 | 3.46 | 0.06 | 94.15 | 97 | 1.53 | 0.22 |
|  |  | TC | 252.02 | 233 |  |  | 58.7 | 53 |  |  |
|  |  | CC | 52.49 | 62 |  |  | 9.15 | 12 |  |  |
|  | **rs10519097** | CC | 424.58 | 422 | 0.58 | 0.45 | 112.5 | 110 | 2 | 0.16 |
|  |  | CT | 167.83 | 173 |  |  | 45 | 50 |  |  |
|  |  | TT | 16.58 | 14 |  |  | 4.5 | 2 |  |  |
| ***TIMELESS*** | **rs774027** | AA | 156.03 | 157 | 0.02 | 0.89 | 40.5 | 44 | 1.21 | 0.27 |
|  |  | AT | 302.93 | 301 |  |  | 81 | 74 |  |  |
|  |  | TT | 147.03 | 148 |  |  | 40.5 | 44 |  |  |
|  | **rs3809125** | CC | 251.82 | 250 | 0.1 | 0.75 | 69.13 | 70 | 0.09 | 0.76 |
|  |  | CT | 276.36 | 280 |  |  | 72.74 | 71 |  |  |
|  |  | TT | 75.82 | 74 |  |  | 19.13 | 20 |  |  |
|  | **rs7302060** | TT | 182.99 | 181 | 0.11 | 0.74 | 52.25 | 55 | 0.78 | 0.38 |
|  |  | TC | 300.03 | 304 |  |  | 79.51 | 74 |  |  |
|  |  | CC | 122.99 | 121 |  |  | 30.25 | 33 |  |  |
