# Supplementary material for: Associations of clock genes polymorphisms with soft tissue sarcoma susceptibility and prognosis
Source: J Transl Med. 2018 Dec 5;16:338. doi: 10.1186/s12967-018-1715-0 (PMC6280400; doi:10.1186/s12967-018-1715-0)
Supplement: Supplementary file 2 — Additional file 2: Table S2. Statistical power for all the SNPs tested in this study in sarcoma patients. [file 12967_2018_1715_MOESM2_ESM.doc]

**Additional file 2: Table S2** *Statistical power for all the SNPs tested in this study in sarcoma patients.*

| **Gene** | **SNP ID** | **MAF (Ctrls)** | **POWER** |
| --- | --- | --- | --- |
| *CLOCK* | rs1801260 | 0.28 | 0.33 |
|  | rs3736544 | 0.38 | 0.39 |
|  | rs3749474 | 0.36 | 0.38 |
| *NPAS2* | rs895520 | 0.41 | 0.40 |
|  | rs2305160 | 0.32 | 0.36 |
| *PER1* | rs3027178 | 0.33 | 0.37 |
| *PER2* | rs934945 | 0.20 | 0.26 |
|  | rs7602358 | 0.24 | 0.30 |
| *RORA* | rs339972 | 0.29 | 0.34 |
|  | rs10519097 | 0.17 | 0.23 |
| *TIMELESS* | rs774027 | 0.49 | 0.43 |
|  | rs3809125 | 0.35 | 0.38 |
|  | rs7302060 | 0.45 | 0.42 |
