# Supplementary material for: Associations of clock genes polymorphisms with soft tissue sarcoma susceptibility and prognosis
Source: J Transl Med. 2018 Dec 5;16:338. doi: 10.1186/s12967-018-1715-0 (PMC6280400; doi:10.1186/s12967-018-1715-0)
Supplement: Supplementary file 3 — Additional file 3: Table S3. Upper panel: associations of circadian pathway genes with prognosis of sarcoma under 3 models of inheritance (additive, recessive, dominant); lower panel: association of circadian pathway genes with prognosis of liposarcoma and leiomyosarcoma in subgroups analysis under the additive genetic model. [file 12967_2018_1715_MOESM3_ESM.doc]

**Additional file 3: Table S3** *Upper panel: associations of circadian pathway genes with prognosis of sarcoma under 3 models of inheritance (additive, recessive, dominant); lower panel: association of circadian pathway genes with prognosis of liposarcoma and leiomyosarcoma in subgroups analysis under the additive genetic model*

| ***Sarcoma*** |  |  |  |  |  |  |  |
| --- | --- | --- | --- | --- | --- | --- | --- |
|  |  | ***Additive*** |  | ***Recessive*** |  | ***Dominant*** |  |
| ***Gene*** | **SNP ID** | **HR [95% CI]** | **P-val.** | **HR [95% CI]** | **P-val.** | **HR [95% CI]** | **P-val.** |
| ***CLOCK*** | rs1801260 | 1.06 [0.71-1.60] | 0.77 | 1.39 [0.50-3.92] | 0.53 | 1.02 [0.62-1.66] | 0.94 |
|  | rs3736544 | 0.95 [0.66-1.37] | 0.76 | 1.41 [0.72-2.73] | 0.31 | 0.75 [0.45-1.23] | 0.25 |
|  | rs3749474 | 1.01 [0.69-1.48] | 0.97 | 1.77 [0.88-3.58] | 0.11 | 0.81 [0.50-1.31] | 0.40 |
| ***NPAS2*** | rs895520 | 1.01 [0.73-1.39] | 0.95 | 0.87 [0.48-1.55] | 0.63 | 1.15 [0.68-1.94] | 0.59 |
|  | rs2305160 | 0.90 [0.63-1.29] | 0.56 | 0.98 [0.48-2.01] | 0.97 | 0.83 [0.51-1.34] | 0.44 |
| ***PER1*** | rs3027178 | 0.98 [0.66-1.44] | 0.91 | 2.09 [0.94-4.63] | 0.07 | 0.79 [0.49-1.26] | 0.33 |
| ***PER2*** | rs934945 | 0.70 [0.41-1.20] | 0.19 |  |  | 0.71 [0.41-1.22] | 0.21 |
|  | rs7602358 | 1.20 [0.82-1.75] | 0.34 | 1.58 [0.70-3.57] | 0.27 | 1.16 [0.72-1.87] | 0.55 |
| ***RORA*** | rs339972 | 1.03 [0.70-1.51] | 0.88 | 1.66 [0.74-3.72] | 0.22 | 0.91 [0.56-1.47] | 0.69 |
|  | rs10519097 | 0.72 [0.43-1.20] | 0.21 | 1.49 [0.20-11.25] | 0.70 | 0.68 [0.40-1.16] | 0.16 |
| ***TIMELESS*** | rs774027 | 0.86 [0.63-1.18] | 0.36 | 0.70 [0.40-1.25] | 0.23 | 0.91 [0.55-1.53] | 0.73 |
|  | rs3809125 | 0.88 [0.63-1.23] | 0.45 | 0.57 [0.27-1.21] | 0.14 | 1.00 [0.62-1.63] | 0.99 |
|  | rs7302060 | 1.10 [0.81-1.51] | 0.54 | 1.08 [0.62-1.87] | 0.78 | 1.21 [0.73-2.01] | 0.46 |
|  | | | | | | | |
|  |  | ***Liposarcoma*** | | ***Leiomyosarcoma*** | |  |  |
|  | **SNP ID** | **HR [95% CI]** | **P-val.** | **HR [95% CI]** | **P-val.** |  |  |
| ***CLOCK*** | rs1801260 | 1.04 [0.51-2.10] | 0.92 | 0.81 [0.49-1.35] | 0.42 |  |  |
|  | rs3736544 | 0.86 [0.47-1.58] | 0.64 | 1.04 [0.65-1.64] | 0.88 |  |  |
|  | rs3749474 | 1.16 [0.59-2.28] | 0.66 | 1.15 [0.74-1.79] | 0.54 |  |  |
| ***NPAS2*** | rs895520 | 0.96 [0.55-1.69] | 0.89 | 0.97 [0.65-1.45] | 0.87 |  |  |
|  | rs2305160 | 0.95 [0.53-1.70] | 0.85 | 0.87 [0.55-1.37] | 0.55 |  |  |
| ***PER1*** | rs3027178 | 0.99 [0.47-2.05] | 0.97 | 1.00 [0.60-1.67] | 0.99 |  |  |
| ***PER2*** | rs934945 | 0.50 [0.17-1.45] | 0.20 | 0.66 [0.34-1.28] | 0.22 |  |  |
|  | **rs7602358** | **1.98 [1.02-3.85]** | **0.04** | 0.95 [0.58-1.54] | 0.83 |  |  |
| ***RORA*** | rs339972 | 1.01 [0.55-1.85] | 0.98 | 1.14 [0.66-1.99] | 0.63 |  |  |
|  | rs10519097 | 0.44 [0.15-1.30] | 0.14 | 0.91 [0.50-1.66] | 0.75 |  |  |
| ***TIMELESS*** | rs774027 | 0.63 [0.35-1.11] | 0.11 | 0.77 [0.51-1.15] | 0.20 |  |  |
|  | rs3809125 | 0.88 [0.48-1.61] | 0.68 | 0.87 [0.56-1.33] | 0.52 |  |  |
|  | rs7302060 | 1.65 [0.93-2.90] | 0.08 | 1.20 [0.78-1.82] | 0.41 |  |  |
